# Discrete-Valued Time Series

**DOI:** 10.3390/e25121576

**Published:** 2023-11-23

**Authors:** Christian H. Weiß

**Affiliations:** Department of Mathematics and Statistics, Helmut Schmidt University, 22043 Hamburg, Germany; weissc@hsu-hh.de; Tel.: +49-40-6541-2779

Time series are sequentially observed data in which important information about the phenomenon under consideration is contained not only in the individual observations themselves, but also in the way these observations follow one another. Therefore, the stochastic counterpart of time series data, the stochastic process, and the methods and models for time series analysis, carefully consider this sequential information. The first approaches to the analysis and modeling of time series were developed about 100 years ago, and they have formed an entire research discipline at least since the publication of the famous textbook by Box and Jenkins [1] about 50 years later. This double anniversary recently gave rise to the Special Issue “Time Series Modeling” in *Entropy* [2], where numerous contributions illustrated the strong research interest and the various research directions in this area. Here, it is interesting to note that these contributions could be roughly split into two halves. The first half deals with real-valued time series, i.e., time series having a continuous range consisting of real numbers or real vectors. Indeed, time series of this kind have been the main subject of time series analysis since the beginning, and for most of the last 100 years. Remarkably, however, the second half of the papers deals with a rather young subfield of time series analysis that now seems to be attracting a great deal of research interest: discrete-valued time series. The first papers on discrete-valued time series appeared in the 1980s, but it was not until the 2000s that a rapid increase in research activity could be observed. It is only in the last few years that a certain maturity and consolidation of this research area can be observed, which is manifested in, among other things, the textbooks by Davis et al. [3], Weiß [4], and in a number of recent survey articles, such as Davis et al. [5], Fokianos [6], Armillotta et al. [7], Karlis and Mamode Khan [8], and Li et al. [9], as well as in contribution 4 by Liu et al.

Discrete-valued time series can be of several types (see Weiß [4] for a comprehensive discussion). Undoubtedly, the most popular are count time series, where the range is quantitative and consists of non-negative integers [5,6,7,8]. However, truly integer-valued time series (where the range also includes negative integers) are increasingly being considered [9], while categorical time series, where the range is qualitative (symbols), are still somewhat neglected. Finally, the discretization of a real-valued time series has to be mentioned in this context, e.g., when methods based on ordinal patterns are used for its analysis (see Bandt [10] for a recent overview). Motivated by these diverse research directions, this Special Issue on “Discrete-valued Time Series” was initiated, which was actually very well received. It was possible to collect articles on a wide range of topics in this area, covering stochastic models for discrete-valued time series, as well as methods for their analysis, univariate and multivariate discrete-valued time series, and various applications of discrete time-series methods. The remainder of this Editorial provides a brief summary of the contributions to this Special Issue, grouping the articles thematically.

A large class of models for count time series make use of so-called thinning operators [4]. Such thinnings are used as integer substitutes of the multiplication, and allow us to adapt the classical autoregressive moving-average (ARMA) models to the count-data case. Therefore, the resulting integer ARMA-type models are abbreviated as INARMA models [4]. The observation-driven random parameter INAR(1) model proposed by Yu and Tao (contribution 1) belongs to this class, and it is characterized by using the Poisson thinning operator together with a state-dependent thinning parameter (i.e., the thinning parameter at time *t* depends on the count observation at time t−1). Furthermore, the authors introduce further flexibility into the model by allowing the thinning parameter to be chosen randomly (given the previous observation). The AR(1)-type model developed by Chen et al. (contribution 2) belongs to the INARMA family as well, but differs from the aforementioned model of Yu and Tao in several respects. First, Chen et al. focus on bounded counts (i.e., the observed counts have the range {0,…,n} with specified n∈N={1,2,…}), whereas Yu and Tao’s INAR(1) model assumes the unbounded range $N_{0}=\left\{0,1,\ldots \right\}$. Second, Chen et al. use Conway–Maxwell–Poisson-binomial thinning operators, thus allowing the resulting model to handle counts exhibiting either underdispersion, equidispersion, or overdispersion with respect to a binomial distribution. Further, the article by Silva et al. (contribution 3) refers to the INARMA modeling of count time series, but under a different perspective. In many application contexts, it is not possible to observe the true count value at a time *t*, but only a censored version of it. Neglecting censoring might cause biased and inconsistent model estimates, so Silva et al. developed two Bayesian algorithms to explicitly account for the censoring of data while fitting an INAR(1) model.

Another large class of models for count time series adapt an ARMA-like structure via a conditional regression approach. Although there is an ongoing debate on how these models should be referred to [4], most authors use the term integer-valued generalized autoregressive conditional heteroskedasticity (INGARCH). Compared to the INARMA class, INGARCH models are better able to handle higher-order models, and by using a feedback term within the model recursion, they also allow for an intensified memory of the count process. For these reasons, among others, INGARCH models have become very popular in recent years, and a comprehensive survey about the state of the art is presented by Liu et al. (contribution 4). It is worth noting that their survey article is not restricted to univariate counts only (where both the cases of unbounded and bounded counts are considered), but it also covers INGARCH models for multivariate counts, as well as models for “Z-valued time series”. The latter refers to time series with range Z={…,−1,0,1,…}, i.e., where also negative integer outcomes are possible (also see Li et al. [9] for further information). At this point, the article by Xu et al. (contribution 5) has to be mentioned, where a particular type of INARCH model is developed. The proposal adapts the multiplicative error model to the count-data case by combining the INARCH approach with the binomial thinning operator. Consequently, the resulting model might be understood as a hybrid model that combines features from the INARMA and INGARCH classes. Finally, the paper of Moontaha et al. (contribution 6) is also concerned with the modeling of (possibly multivariate) count time series, but the proposed approach does not fall into either the INARMA or the INGARCH class. On the contrary, although this may seem contradictory at first, Moontaha et al. use the Gaussian linear state space model together with the Kalman filter. However, to ensure that non-negative integer outcomes are generated, the model is equipped with special observation functions.

The remaining contributions to this Special Issue are not about the mere modeling of discrete-valued time series, but consider miscellaneous topics in this research area. In Weiß et al. (contribution 7), a particular type of analyzing count time series is discussed, namely hypothesis tests based on the partial autocorrelation function (PACF). Such PACF tests are commonly used for identifying the model order of AR-type count processes, but their asymptotics have been derived for Gaussian data. Therefore, different ways of getting more reliable test implementations are investigated in a comparative study. Guan and Wang (contribution 8) present an application of INARMA models to the risk analysis of an insurance portfolio. More precisely, an INAR(1) model is used to describe the temporal dependence among the premium numbers, and an INMA(1) model for the temporal dependence among the claim numbers. Another application of INARMA models is presented by Morais (contribution 9), namely statistical process monitoring by control charts. Morais derives two stochastic ordering results regarding the geometric INAR(1) process which, in turn, can be utilized to conclude on the properties of the geometric control chart’s run length distribution. Last but not least, Papapetrou et al. (contribution 10) discuss the causality analysis of discrete-valued multivariate time series. In contrast to the aforementioned contributions, these time series do not need to consist of quantitative count values, but might also be of qualitative nature. Papapetrou et al. propose a discrete type of partial mutual information from mixed embedding, and investigate its performance in a simulation study.
